# Coronal Cementum and Reduced Enamel Epithelium on Occlusal Surface of Impacted Wisdom Tooth in a Human

**DOI:** 10.3390/dj12110348

**Published:** 2024-10-30

**Authors:** Naohiro Horie, Masaru Murata, Yasuhito Minamida, Hiroki Nagayasu, Tsuyoshi Shimo, Toshiyuki Akazawa, Hidetsugu Tsujigiwa, Youssef Haikel, Hitoshi Nagatsuka

**Affiliations:** 1 Division of Reconstructive Surgery for Oral and Maxillofacial Region, School of Dentistry, Health Sciences University of Hokkaido, Tobetsu 061-0293, Japan; horien@hoku-iryo-u.ac.jp (N.H.); shimotsu@hoku-iryo-u.ac.jp (T.S.); 2 Division of Regenerative Medicine, School of Dentistry, Health Sciences University of Hokkaido, Tobetsu 061-0293, Japan; 3 Division of Oral and Maxillofacial Surgery, School of Dentistry, Health Sciences University of Hokkaido, Tobetsu 061-0293, Japan; minamida@hoku-iryo-u.ac.jp (Y.M.); nagayasu@hoku-iryo-u.ac.jp (H.N.); 4 Industrial Technology and Environment Research Development, Hokkaido Research Organization, Sapporo 001-0021, Japan; akazawa-toshiyuki@hro.or.jp; 5 Department of Life Science, Faculty of Science, Okayama University of Science, Okayama 700-0005, Japan; 6 Department of Biomaterials and Bioengineering, Institut National de la Santé et de la Recherche médicale Unité Mixte de Recherche (INSERM UMR) _S 1121, University of Strasbourg, 67000 Strasbourg, France; youssef.haikel@unistra.fr; 7 Department of Oral Pathology and Medicine Faculty of Medicine, Dentistry and Pharmaceutical Sciences, Okayama University, Okayama 700-8530, Japan; jin@okayama-u.ac.jp

**Keywords:** coronal cementum, human, reduced epithelium, impacted tooth

## Abstract

**Background:** There is only limited research on the coronal cementum of a tooth, and the mechanisms of its forming process are not well-defined. This report presents a coronal cementum on the occlusal surfaces of enamel in an impacted wisdom tooth in a human, which is not nearly the cervical portion. **Materials and Methods:** The tooth (Tooth #1) was derived from a 46-year-old female. Histological analysis, including hematoxylin and eosin (HE) and toluidine blue (TB) staining, and Scanning Electron Microscopy and Energy Dispersive X-ray Spectrometer (SEM-EDS) analysis of the extracted tooth were conducted. Radiographic examination showed that Tooth #1 was horizontally impacted in the maxilla and had the apex of a single root placed between the buccal and palatal roots of Tooth #2. **Results:** Coronal cementum was distributed widely on the enamel, and reduced enamel epithelium was also found with enamel matrix proteins histologically. The formation of acellular cementum was observed to be more predominant than that of the cellular cementum in Tooth #1. SEM showed that the occlusal cementum connected directly with enamel. Calcium mapping revealed an almost similar occlusal cementum and enamel. In addition, the spectrum of elements in coronal cementum resembled the primary cementum according to SEM-EDS. **Discussion:** Thus, coronal cementogenesis in impacted human teeth might be related to the existence of reduced enamel epithelium.

## 1. Introduction

Cementum is a hard tissue composed of water, minerals, and organic minerals, including type I collagen with type III collagen [1]. The mechanism of cementum development is as follows. At the onset of root dentin formation, Hertwig’s epithelial sheath degenerates and breaks off, and undifferentiated mesenchymal cells derived from dentinal tubules pass through the gap and come into contact with the root dentin surface. The undifferentiated mesenchymal cells of root dentin change into cementoblasts, which secrete a cementitious material. Cementoblasts migrate while secreting cementitious organelles, and those cells that are completely surrounded by cementitious organelles are called cement cells. Cementum is classified into cellular or acellular cementum according to the presence of cementocytes and fiber or afibrillar cementum according to the existence of collagen fiber. Cellular cementum, containing cementocytes, is mainly distributed near the apex of the root, and acellular cementum is observed between the dentin and periodontal ligaments. Coronal cementum was first reported in the late 17th century (1691) as a cementum directly covering the enamel at horse molars in “Osteologia Nova” by Havers. Since then, coronal cementum has been reported in some animal species, including humans [2,3,4]. The microstructure of coronal cementum in horse teeth has shown the laminated structure in the coronal cementum, which may have been formed by cementoblasts and Sharpey’s fiber insertion into the cementum [3]. Also, specimens of cattle tooth germs showed that the formation of coronal cementum had initiated in the bottom area of occlusal grooves [4]. Meanwhile, the microradiographic observation of coronal cementum at an occlusal fissure on an unerupted human teeth was published in 1976 [5]. And in 2009, Ho et al. reported the structure, chemical composition, and mechanical properties of human coronal cementum on the cement–enamel junction at the cervical position [6]. However, histological estimation of occlusal cementum and reduced enamel epithelium is extremely rare in humans, but not in herbivorous mammals, such as bovines and horses [7]. There were also no reports on the human coronal cementum from 2022 to 2024. Notably, the mechanism of cementogenesis is unclear at the coronal portion of the human tooth. In the present study, we focus on the histological findings and Scanning Electron Microscopy and Energy Dispersive X-ray Spectrometer (SEM-EDS) analyses of coronal human cementum formed on the occlusal surfaces of enamel in a completely impacted wisdom tooth in the maxilla. The tooth numbers were assigned by following a universal system. In addition, this research was approved by the ethics committee at the Hearth Science University of Hokkaido (Approval No. 2).

## 2. Materials and Methods

### 2.1. Patient

**Figure 1:**
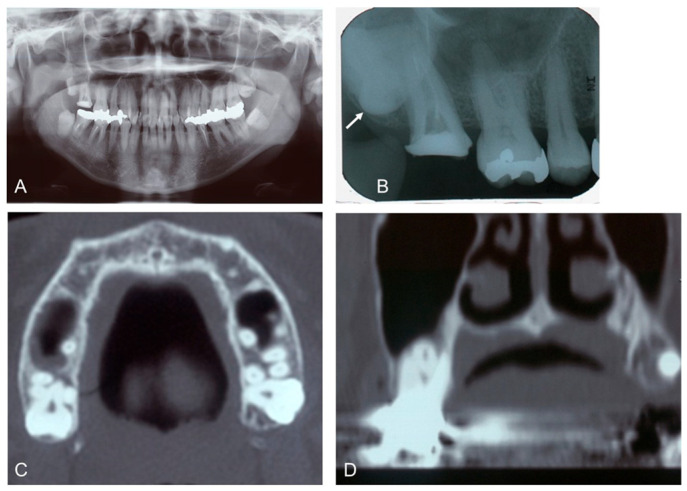
Initial X-ray images of the 46-year-old female patient. (**A**): Panoramic image. Note: impacted wisdom teeth (#1, 16, 17, 32). (**B**): Dental image (#1, 2, 3, 4). Arrow indicates horizontally impacted tooth (#1). (**C**): CT (axial image). (**D**): CT (coronal image) showing root apex of upper right third molar (#1) placed between buccal and palatal roots of upper right second molar (#2).

A 46-year-old female revealed a fistula at the apex of the palatal root of the upper right second molar (Tooth #2). She had a history of non-tuberculous mycobacteriosis, Hashimoto’s thyroiditis, and submucosal tumor of the large intestine. Clinical examination required the extraction of the upper right third molar (Tooth #1) and the palatal root of Tooth #1. Radiographic examination showed that the wisdom tooth (Tooth #1) was horizontally impacted in the maxilla and had the apex of a single root placed between the buccal and palatal roots of Tooth #2 (Figure 1).

### 2.2. Surgical Procedure

The palatal root of Tooth #2 was resected by a diamond bar and extracted by forceps under local anesthesia. Next, the impacted Tooth #1 was extracted after elevation of the mucoperiosteal flap. The crown portion of Tooth #1 was covered with hard tissues.

### 2.3. Light Microscopy and Topographical Analysis by SEM-EDS

The extracted Tooth #1, which was fixed in 10% neutral phosphate-buffered formalin solution for 1 week, was divided into two samples by a diamond cutter (Morita, Tokyo, Japan). One half of the sample was decalcified with 10% formic acid for 4 weeks and rinsed overnight with running water. After dehydration using 50–100% ethanol, the sample was processed for paraffin embedding (Vacuum Rotary, Mitsubishi, VRX-23, Tokyo, Japan). The paraffin block was sliced into sections using a microtome (Yamato ROM-380, Tokyo, Japan), and they were stained with hematoxylin and eosin (HE) (Wako, Osaka, Japan). The other undecalcified sample was embedded in epoxy resin (Nissin EM, NER-814, Tokyo, Japan), and after the polishing, histological sections were prepared by a hard-tissue microtome (LEICA, SP1600, Wetzlar, Germany) for toluidine blue (TB) staining and SEM-EDS analyses. SEM (HITACHI, S-3500N, Tokyo, Japan) images of the non-demineralized sample (#1) were taken to observe the morphology and microstructure. At the same time, characteristic X-ray spectra of mineral components in the surface region (about 1 μm depth) of tooth were measured at an accelerating voltage of 10.0 kV using EDS (HITACHI, S-3500N, Tokyo, Japan). From the EDS spectra, residual contents of different elements for a normal root and coronal cementum were calculated, and the Ca-concentration distribution was shown in an X-ray image for the enamel and coronal cementum regions.

## 3. Results

### 3.1. Gross View

**Figure 2:**
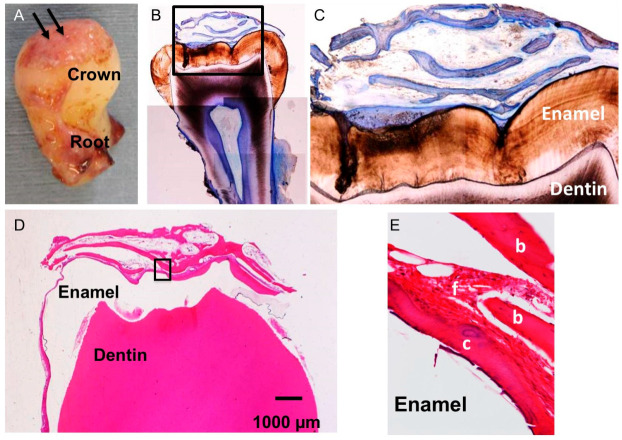
Whole image and histological views of extracted Tooth #1. (**A**): Macroscopic image of #1 showing crown covered with hard tissues. Arrows indicating hard tissues. (**B**): Non-demineralized section stained with TB. Hard tissues (blue color) and enamel (brown) in black line area. (**C**): Higher magnification of the black line area in B showing enamel surface covered directly with hard tissues. Blue-stained hard tissues over brown-stained enamel. (**D**): Demineralized section stained with HE showing lower magnification of crown region. Clear white space indicates completely demineralized enamel. (**E**): Higher magnification of black line box in (**D**) showing cementum attached with enamel. Note: Cementum distinguished from bone, including osteocytes. b: bone, c: cementum, and f: fibrous connective tissue.

The crown of Tooth #1 was covered with hard tissues (Figure 2A).

### 3.2. Histological Observation

**Figure 3:**
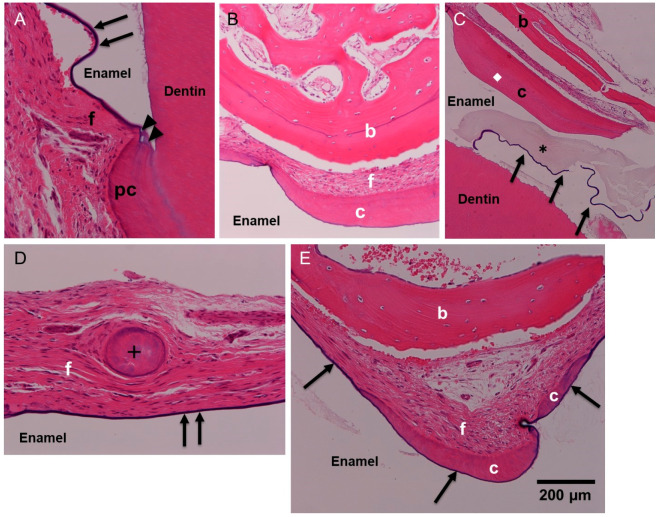
Histological views of horizontal impacted Tooth #1 in HE. (**A**): Arrows indicating reduced enamel epithelium showing thin bundle. Note: Attachment to primary cementum (PC)–enamel (white clear space) junction (arrowhead). (**B**): Occlusal cementum (c) on enamel. (**C**): Co-existence of acellular and cellular cementum matrix. Enamel protein (*) near reduced enamel epithelium (arrows). (**D**): Cross mark indicating detached cementum pearl in fibrous connective tissues. (**E**): Acellular coronal cementum contacted directly with reduced enamel epithelium on enamel. Bone with osteoblast lining. b: bone, c: cementum, f: fibrous connective tissue, pc: primary cementum, *: enamel matrix protein, ◆: cellular cementum, arrow: reduced enamel epithelium, arrowhead: primary cementum–enamel junction, cross mark: cementum pearl.

The occlusal surface grooves of enamel were covered directly with TB-stained hard tissues (Figure 2B,C). The hard tissue onto enamel was distinguished from trabecular bone including osteocytes and looked like cementum (Figure 2E and Figure 3B,C,E). The reduced enamel epithelium showed a violet bundle in HE and was attached to the primary cementum–enamel junction (Figure 3A). In the histological structure, four layers (enamel, occlusal cementum, fibrous tissue, and bone) were observed (Figure 3B,E). Furthermore, the residue of enamel matrix protein and reduced enamel epithelium were found near the coronal cementum (Figure 3C). Interestingly, a cementum pearl was seen near the reduced enamel epithelium on the enamel (Figure 3D). The four-layer structure was confirmed, and coronal cementum had especially contacted the reduced enamel epithelium on the enamel directly (Figure 3E).

### 3.3. SEM-EDS Analyses

**Figure 4:**
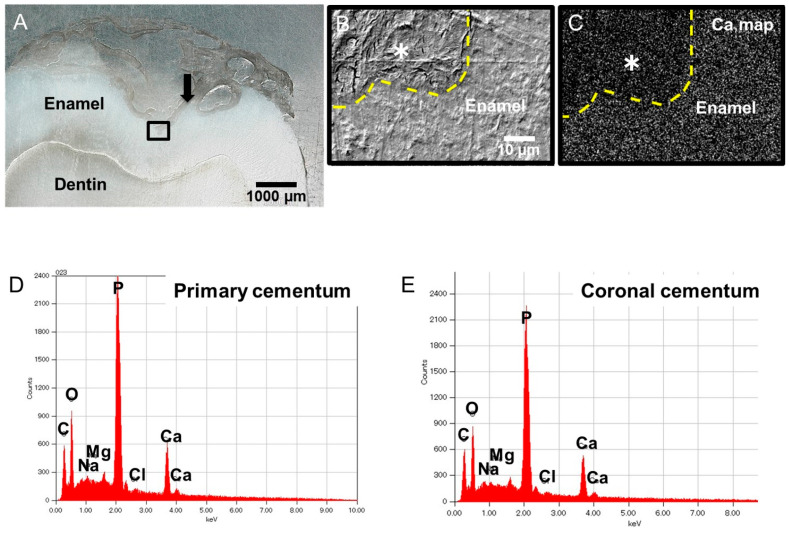
SEM-EDS analyses. (**A**): SEM image of crown region of extracted tooth. (**B**): High-magnification image of black box area in A. (**C**): Tiny white dots indicate distribution of Ca element. Yellow dotted line shows border between coronal cementum (*) and enamel. (**D**,**E**): Spectrum of EDS analysis. (**D**): Primary cementum. (**E**): Coronal cementum pointed out by arrow in A.

SEM showed the occlusal cementum connected directly with enamel (Figure 4A,B). Calcium mapping revealed almost similar occlusal cementum and enamel (Figure 4C). To evaluate the quality of coronal and primary cementum, the spectrum of elements was quantitatively assessed by EDS. The spectrum of the elements (calcium, chlorine, phosphorus, magnesium, sodium, oxygen, and carbon) in the coronal cementum resembled that in the primary cementum (Figure 4D,E).

## 4. Discussion

We discovered human coronal cementum histologically on the occlusal surfaces of enamel in a completely impacted wisdom tooth in the maxilla, which is not near the cervical portion. Interestingly, the coronal cementum in Tooth #1 was distributed widely in a range of approximately 5 mm and consisted predominantly of acellular and cellular cementum. 

Generally, coronal human cementum was found as a thin layer of acellular cementum adjacent to enamel in the cervical portion [6]. The structure, chemical composition, and mechanical properties of cervical coronal cementum in human mandibular molars were investigated at the junction between the coronal cementum and enamel. The results showed that coronal cementum consisted of acellular afibrillar and acellular fiber cementum, the junction appeared be to integrated micromechanically via a weak interface with enamel, and the elastic modulus of the coronal cementum was significantly lower than that of the primary cementum [6]. The presence of coronal cementogenesis was reported on the outer surface of the enamel organ or the reduced enamel epithelium [8]. We consider enamel matrix proteins as one of the key factors of coronal cementogenesis. A porcine enamel matrix protein derivative is clinically available for periodontal regeneration. A basic study in monkey showed that mesenchymal cells of dental follicles exposed to enamel matrix proteins formed acellular cementum on the enamel surface [9]. On the other hand, cellular cementum was observed in a buccal dehiscence model without an enamel matrix [10]. We hypothesize the mechanism of coronal cementogenesis to have the following steps: 1. invasion of mesenchymal cells of dental follicles from the denatured, opened area of reduced enamel epithelium and 2. metaplasia of the reduced enamel epithelium.

In the present study, HE sections showed reduced enamel epithelium continuously from the cement–enamel junction, a broken part of the reduced enamel epithelium, acellular and cellular cementum on the reduced enamel epithelium, a free cementum pearl, and residues of enamel proteins. Morphologically, four layers (enamel, coronal cementum, fibrous connective tissue, and bone) were observed clearly. EDS analysis showed that the composition of the coronal cementum was almost similar to that of the primary cementum. The Ca/P ratio of the coronal cementum might have been slightly lower than that of the primary cementum, while the platinum used by vapor deposition overlapped the phosphate (P) ion peak. 

Based on our histological findings, we believe that the enamel proteins were able to differentiate undifferentiated mesenchymal cells derived from the dental follicles into cementoblasts, which produced acellular and/or cellular cementum on the enamel and a free cementum pearl in the fibrous connective tissues. A further hypothesis derived from the above is that this mechanism of the coronal cementum formation is induced during the development of impacted teeth.

Coronal cementum on the occlusal surfaces of enamel was found histologically in the impacted human wisdom tooth. The formation of acellular cementum was observed to be histologically more predominant than that of the cellular cementum in Tooth #1. Coronal cementogenesis in impacted human teeth might be involved in the existence of reduced enamel epithelium. Our case might be the first histological report related to both acellular and cellular matrices of coronal cementum and the reduced enamel epithelium in humans and not in herbivorous mammals, such as bovines and horses. The number of reports on coronal cementum on human teeth are limited, and our data were obtained from one patient’s specimen. Therefore, it is undeniable that this report may have a selective bias, and additional findings are required in order to elucidate the developmental biology of human coronal cementum.

After extracting impacted teeth, the dentist checks the entirety of the extracted tooth (root morphology, number, and existence of fractures). Typically, the enamel of the extracted tooth is exposed, and the morphology of the cusp can be seen. On the other hand, this sample was of scientific interest in that the cuspal morphology of the crown was not visible and was covered by hard tissue; hence, histological and SEM-EDS analyses were attempted. Since there are few reports on the coronal cementum, the results obtained in this study provide valuable data for the differential diagnosis of tooth-related diseases (such as odontogenic tumors). And we would like to emphasize the academic potential of the extracted tooth, which would normally be discarded.

## Data Availability

The data presented in this study are available on request from the corresponding author due to privacy of the patient.
